# Retraction: QKI5mediated alternative splicing of the histone variant macroH2A1 regulates gastric carcinogenesis

**DOI:** 10.18632/oncotarget.28866

**Published:** 2026-04-17

**Authors:** Feng Li, Ping Yi, Jingnan Pi, Lanfang Li, Jingyi Hui, Fang Wang, Aihua Liang, Jia Yu

**Affiliations:** ^1^Institute of Biotechnology, Key Laboratory of Chemical Biology and Molecular Engineering of National Ministry of Education, Shanxi University, Taiyuan, PR China; ^2^Department of Biochemistry, Institute of Basic Medical Sciences, Chinese Academy of Medical Sciences (CAMS) and Peking Union Medical College (PUMC), Beijing, PR China; ^3^Department of Obstetrics and Gynecology, Research Institute of Surgery, Daping Hospital, Third Military Medical University, Chongqing, PR China; ^4^State Key Laboratory of Molecular Biology, Shanghai Institutes for Biological Sciences, Chinese Academy of Sciences, Shanghai, PR China

**This article has been retracted:** An investigation by the Oncotarget Scientific Integrity office has concluded. The investigation identified multiple instances of image manipulation and duplication with other published works. Some of these comparative articles share two authors with this paper (articles [1] and [5]) or one author (articles [6] and [7]), published earlier or later, respectively.

The specific duplications and manipulations include:

- Figure 2E: Transwell assay/HGC-27 cells images are duplicates of control images in Figure 3D of [1], Figure 2B of [2], Figure 2D of [3], and Figures 6A/B of [4]. Additionally, the Transwell assay/MGC-803/pcDNA image overlaps with Figure 5D/vii of [5].
- Figure 3D: The authors stated that the IHC staining images for Ki-67 and caspase-3 in the Control group were selected in error.
- Figure 5D: The Wound Healing assay image is a duplicate of the image in Figure 4F of [3].
- Figure 5E: The Transwell assay/HGC-27 cells invasion image in the pcDNA group overlaps with the TOX+LY image in Figure 7D of [4].
- Figure 5F: In the IHC H&E staining, panels “normal” #10 and #26 were reused in Figure 4D of [6] as “normal” #3 and #25. The cancer H&E staining #1 appears to be reused in Figure 3D of retracted article [7].

Authors Feng Li and Jia Yu provided some explanation and initially requested a correction. They attributed the misuse of images to similar experiments performed by Feng Li, Lihua Guo, and Yuxuan Wang in a shared laboratory environment; however, the duplications with other earlier-published papers were not satisfactorily explained. Furthermore, the authors did not provide original data to support the requested corrections.

Due to the substantial number of confirmed duplications and overlaps, combined with the absence of original supporting data, the Editorial decision to retract this paper has been made. While the authors eventually agreed to the retraction, they did not provide signed agreement from all listed authors.

Original article: Oncotarget. 2016; 7:32821–32834. 32821-32834. https://doi.org/10.18632/oncotarget.8739
